# Food insecurity in the household: Interventions tackling children’s food insecurity, a commentary on a systematic review

**DOI:** 10.12968/jfch.2024.1.3.118

**Published:** 2024-11-02

**Authors:** J Harrison, G Allen, A Clegg

**Affiliations:** University of Lancashire; Public Advisor, NIHR Applied Research Collaboration North West Coast; University of Lancashire

**Keywords:** Household food insecurity, hunger, Holiday Activity and Food programmes, children’s health, nutrition, education

## Abstract

In the UK, 7% of households are deemed food insecure, that is they are unable to access, acquire and prepare food for the table. Food insecurity is associated with numerous negative impacts for children such as a reduced nutritional intake, social, behavioural and developmental problems and reductions in academic or cognitive performance. Poorer mental health outcomes are also experienced by both children and parents living with food insecurity. A systematic review of interventions to tackle children’s food insecurity was undertaken by Holley et al. (2019). This commentary critically appraises the review and discusses what the findings imply for the provision of such interventions, particularly those related to holiday, activity and food clubs.

## Introduction

Household food security or consistent access to healthy food, is determined by the ability to afford food, physical access to buy food, and the opportunity to prepare nutritious meals (DEFRA 2021). According to government data from the financial year ending 2022, 7% of households in the UK do not consistently meet these three tests and are defined as food insecure (DEFRA 2024). Numerous factors can have an impact on household food security including age, disability, ethnicity, and geographical location (DEFRA 2021). In the UK, the North-East and West Midlands have the highest levels of food insecurity (10 and 9% respectively), and the East, South-west and London all have the lowest levels at 5% (DEFRA 2024).

Food insecurity is a constant factor in the lives of low-income families in the UK even with initiatives such as free school meals (Shinwell & Defeyter 2021). Negative impacts associated with household food insecurity include child developmental risk, behavioural problems, reduction in school readiness, social, emotional and academic problems and adverse childhood experiences from infancy to adolescence (de Oliveira 2020, Jackson et al. 2021, Royer et al. 2022, Shankar et al. 2017). Nutritionally, there is evidence for a strong and dose-responsive relationship between lower vegetable uptake and higher sugar intake among children experiencing food insecurity (Eicher-Miller et al. 2018). Also, there is a compromised intake of micronutrients such as vitamin D and magnesium (Jun et al. 2021). Transitioning between food security and food insecurity has a significant and lasting effect on cognitive function and externalising behaviour (Gallegos et al. 2021, Grineski et al. 2018). In addition, food insecurity is significantly associated with poorer mental health outcomes in both parents and children (Cain et al. 2022). The presence of food insecurity may also be associated with symptoms of Attention Deficit Hyperactivity Disorder (ADHD) in children, although more research is needed to confirm the relationship (Lu et al. 2019).

Parents experiencing food insecurity may sacrifice their own nutritional needs by buying food that only their children would eat, skipping meals often and eating children’s leftovers (Shinwell & Defeyter 2021). The Covid-19 pandemic increased the needs of these parents further, driven by income reductions and crises, and intensifying food access challenges and benefits (Pautz & Dempsey 2022). Effective public health and policy interventions are evidently required to address the rising prevalence of food insecurity and mental health problems (Cain et al. 2022). A review by Holley & Mason (2019) was undertaken to synthesise the evidence for interventions designed to tackle children’s food insecurity. This commentary aims to critically appraise the methods used within the review and expand upon the findings further in the context of providing and planning relevant interventions.

## Methods of the review by Holley & Mason 2019

This systematic review conducted electronic searches in three electronic databases up to July 2018. The search strategy utilised key-terms relating to ‘food insecurity’ interventions and children or young people. Studies were included in the review that sought to tackle food insecurity and measured the impact of these interventions using outcomes for children. Studies were required to take place in a developed country and be published in a peer-reviewed journal. Studies were excluded if they had a sole focus on the uptake of the intervention (process evaluation) or if outcomes included households or families, rather than children. Screening of included studies, quality assessment and data extraction using a standardised form was undertaken by one author. Quality assessment was undertaken using the Mixed Methods Appraisal Tool (MMAT). Outcomes assessed were either those that looked at the effectiveness of the intervention in increasing the consumption of food types or those that examined the impact on the children themselves (e.g., behaviour, educational achievement).

## Results of Holley & Mason 2019

Forty-two studies were included in the systematic review with the majority taking place in the US (34 studies) and the remainder taking place in the UK (4 studies), Australia, Canada, Greece and New Zealand (all 1 study). Interventions were reported into two categories, ‘attended’ (in person) or ‘subsidy’ interventions. For the attended intervention studies 79% met all five quality criteria of the MMAT and 83% met three. Of the subsidy interventions studies, 50% met all five quality criteria and 95% met at least three.

### How do studies evaluate food insecurity?

Most studies included in the review were non-randomised quantitative studies (n=32), using experimental and observational designs, estimating the effectiveness of an intervention. The review reported that whilst the majority were well conducted, areas that were lacking methodologically were the accounting for confounding factors in the study design/analysis and incomplete outcome data, with few reporting the duration of the intervention or participation level. The low number of randomised controlled trials (RCTs) identified (n=5) was likely to avoid withholding an intervention designed to help children. Consequently, using a non-randomised design to compare similar individuals may have led to a selection bias in participants. Experimental studies were low quality, small scale and with outcomes limited to parent or self-report measures. Three studies were qualitative and two were mixed methods. Qualitative studies predominantly reported parent and stakeholders’ views rather than children.

### Outcomes for attended interventions

Of the 24 ‘attended’ intervention papers included in the review, thirteen related to school food assistance that provided breakfast and lunch provision. In a Canadian study, participation in school food assistance was found to improve educational difficulties for children experiencing household food insecurity. In the US, a study found that participation resulted in a reduced risk of obesity for food insecure girls, but not for boys, and in another study, didn’t increase the risk of obesity for food insecure children. Also in the US, school-based food assistance was shown to reduce the odds of children experiencing food insecurity amongst high-risk border populations. Breakfast interventions specifically were found to reduce food insecurity in two US studies. In the UK, universal free school breakfast were perceived to help alleviate hunger, improve health outcomes and provide social, behavioural and educational benefits. Evidence for academic performance was mixed with one US study reporting no evidence and a UK study reporting improved concentration and less skipped classes. Participation in lunch programmes based in the US showed less favourable health and behaviour outcomes for children in one study, which on further exploration may have been due to uncontrolled familial factors. Another study that controlled for methodological issues such as self-selecting participation, found that a lunch programme did reduce poor health and obesity. Evidence for a relationship between lunch programmes and obesity is mixed however, with one study finding no significant relationship.

Holiday clubs offering free food and enrichment activity for children such as physical activity and crafts were evaluated in three qualitative studies. Findings in the two UK studies suggested reduced hunger and nutritional, social and financial benefits. A US study suggested that parents valued holiday clubs including the opportunity for children to socialise. For interventions that provided education and guidance on nutrition and healthy eating, four out of six studies from the US reported positive outcomes such as children’s consumption of healthy and fresh food, fruit and vegetables, levels of physical activity, willingness to try different foods and intention to change health behaviour. In the two experimental intervention papers based in the US and New Zealand respectively, there was no significant effect on healthy consumption or nutritional intake, but one study reported issues with acceptability and in the other study, children did consume significantly less processed snacks post intervention. Although a comparative study of a programme in the US providing education in addition to a subsidy found no significant change in children’s food insecurity, it was thought to reflect issues with the study design. A US community gardening intervention increased the number of children eating vegetables several times a day but did not change the number of meals missed.

### Outcomes for subsidy interventions

Of the 24 ‘subsidy’ intervention papers included in the review, four were targeted at improving the nutritional health of low-income women and children in the US. They identified that such interventions may reduce the prevalence of food insecurity in children and suggest benefits to health, growth and nutrition. Fourteen further papers explored US based supplemental nutrition assistance programmes (SNAP), providing food purchasing assistance to low-income families. Participation was found to improve educational attainment and reduce poor health amongst food insecure children. Whether participation in SNAP reduced weight status or food insecurity is unclear with studies identifying conflicting results. In two other subsidy provision interventions, one reported lower food insecurity and moderate improvements to healthy consumption whereas the other had no effect. Discounted fruit and vegetables and provision of infant formula did not evidence clear benefit of a positive intervention effect in two further US studies. Reimbursement to child-care providers for meals and snacks in the US resulted in moderately increased consumption of vegetables and milk.

## Commentary

### Critical Appraisal of Holley & Mason 2019

Using the Joanna Briggs Institute Critical Appraisal Tool for Systematic Reviews and Research Syntheses (JBI 2017), four of the 11 criteria were judged to be unclear for the systematic review (Holley and Mason 2019):

▪ There was no narrative regarding evidence of hand-searching in the search strategy. As not all relevant studies are included in electronic databases, hand searching helps to identify those that might be missed (Armstrong et al 2005).
▪ There is an acknowledgement that some aspects of the review including study selection, data extraction and the quality assessment, were undertaken by only one author. Having more than one person in these processes helps to minimise errors and reduce potential biases introduced by review authors (Li et al 2023).
▪ The likelihood of publication bias was not assessed. Potentially, this could mean that some studies were missed, which could cause the effect estimates to be skewed (Page et al 2023).

For these reasons, the review findings should be interpreted with some caution when considering the implications for service provision of food insecurity interventions.

### Provision of food insecurity interventions

The findings of the review were equivocal, partly reflecting the poor quality of the evidence base, the lack of clarity around the interventions and the outcome measures used. The evidence presented suggests that interventions for food insecurity may provide positive health, nutrition and educational outcomes for children and reduce the prevalence and impact of food insecurity. Breakfast clubs were perceived to have more favourable outcomes than lunch clubs and a further Australian study has since reported the social benefits of school-based breakfast clubs including social eating, relationship building and school engagement (Jose et al. 2020). The results of the review suggested that holiday clubs may provide nutritional support including reduced hunger, and increased socialisation for children, whilst helping to ease financial pressure for parents. In the UK, local authorities offer all school-aged children in receipt of benefits related free school meals, participation in the government funded Holiday Activities and Food (HAF) programme, providing healthy meals, enriching activities, and free childcare places during holiday periods (DfE 2024). Local authorities have discretion to use 15% of their funding on free or subsidised places to children who are not in receipt of free school meals but who they believe could benefit from a place (DfE 2024). The HAF Literature Review completed by the UK Department of Education in 2020 was unable to draw substantive conclusions from the evidence about best practice or value for money in holiday food and activity delivery, likely due to lack of evaluation at the time (DfE 2020). More recently, evaluations have shown high demand for HAF, with children taking part being more confident, more likely to participate in sport and exercise, socially connected and improving the quality of children’s dietary behaviours (Crilley et al. 2021, Cox et al. 2022). A HAF programme in Birmingham, UK was evaluated and found to deliver positive outcomes across a wide range of factors including childcare, food security, health and well-being, school readiness, and anti-social behaviour at both an individual, community and city level (Defeyter et al. 2022a). Furthermore, children attending clubs have enjoyed the range of activities provided with attendance improving self-confidence, developing new skills and having opportunities to socialise in a safe environment with peers without stigmatisation (Shinwell et al. 2021).

As school holidays approach, evidence has identified how low-income parents begin provisioning, storing food and reducing household expenditure with acquisition habits becoming more intense during the school holidays (Shinwell & Defeyter 2021). The HAF programme may provide support to low-income parents such as food lasting longer at home and less intense pressure in their approach to food shopping once school resumed. (Shinwell & Defeyter 2021). HAF programmes support childcare demands and allow a quarter of parents to maintain the same working hours or stay in work (Cox et al. 2022). Importantly, parental well-being improved after children attended holiday clubs and reducing social isolation for parents is likely a hidden function of holiday clubs (Long et al. 2021).

### Challenges for the provision on food insecurity interventions

The delivery of interventions designed to tackle food insecurity is not without issue. Challenges for the program delivery of breakfast clubs have included sourcing food, resource limitations, and reliance on volunteers (Jose et al. 2020). Staff working in holiday clubs have highlighted the following as requiring development: staffing considerations (having enough staff), open communication between all the agencies involved, sufficient planning time prior to the club commencing, and more work to encourage attendance of the families who would benefit the most (Graham et al. 2016). The recommended delivery of four hours per day, four days a week for four weeks is seen as rigid for some providers who struggled to meet this level of delivery (Defeyter 2022b). At the local authority level, the increased scope and scale of HAF is viewed as highly complex, involving multiple departments and partners although funding is acknowledged to improve the quality and reach of holiday programmes (Defeyter 2022b). Food menus for holiday clubs may be an area for improvement given that on average they only adhere to school food standards 70% of the time and there is a tendency for food provision to appear less than ideal for attendees aged 11-18 (Vitale et al. 2023).

### Implications for further research

A key finding from Holley and Mason (2019) was a lack of consistent measures and methods in the evaluation of interventions designed to tackle food insecurity. Variation in measurements/methods used to define food insecurity and child development outcomes and the difficulty this poses for making comparisons across studies has been identified elsewhere (Gallegos et al. 2021). The review authors recommend that future research seeks a systems-based approach to implementation and evaluation of these interventions, utilising standardised and universal outcome measures and evidencing how and why such interventions will work. They also recommend that future interventions incorporate the views of families experiencing food insecurity into intervention designs. Evaluations should adopt rigorous designs, clearly defining the intervention evaluated and use consistent outcome measures to assess effectiveness.

## Reflective Questions

What resources are required to run breakfast, lunch or holiday, activity and food clubs?

How can volunteers for such clubs be supported?

How can children’s views be heard in future research of food insecurity interventions?

## Key Points

▪ There is mixed evidence that food insecurity interventions attended by children may have positive outcomes for children including reductions in food insecurity, hunger and obesity, improvement of educational difficulties, and nutritional, social and financial benefits.
▪ Subsidy interventions involving financial assistance may help reduce food insecurity, and improve health and educational outcomes, although evidence is often conflicting.
▪ The evidence available in the review lacked robust evaluation, consistent methods and measures. Qualitative research lacked the views of children, instead focussing on parents and stakeholders.
▪ More recent evidence suggests that Holiday Activity and Food programmes may improve children’s health, social and dietary behaviours. There may also be benefits for parents including less pressure on food provision, support for childcare demands and improvements to well-being.
